# Characterization of Problematic Alcohol Use Among Physicians: A Systematic Review

**DOI:** 10.1001/jamanetworkopen.2022.44679

**Published:** 2022-12-09

**Authors:** Janet Wilson, Peter Tanuseputro, Daniel T. Myran, Shan Dhaliwal, Junayd Hussain, Patrick Tang, Salmi Noor, Rhiannon L. Roberts, Marco Solmi, Manish M. Sood

**Affiliations:** 1 Faculty of Medicine, University of Ottawa, Ottawa, Ontario, Canada; 2 The Ottawa Hospital Research Institute, Ottawa, Ontario, Canada; 3 ICES, Ontario, Canada; 4 Department of Medicine, The Ottawa Hospital, Ottawa, Ontario, Canada; 5 Bruyere Research Institute, Ottawa, Ontario, Canada; 6 Clinical Epidemiology Program, Ottawa Hospital Research Institute, Ottawa, Ontario, Canada; 7 Department of Family Medicine, University of Ottawa, Ottawa, Ontario, Canada; 8 School of Epidemiology and Public Health, University of Ottawa, Ottawa, Canada; 9 Department of Psychiatry, University of Ottawa, Ontario, Canada; 10 Deptartment of Mental Health, The Ottawa Hospital, Ottawa, Ontario, Canada; 11 Department of Child and Adolescent Psychiatry, Charité Universitätsmedizin, Berlin, Germany

## Abstract

**Importance:**

Problematic alcohol use in physicians poses a serious concern to physicians' health and their ability to provide care. Understanding the extent and characteristics of physicians with problematic alcohol use will help inform interventions.

**Objective:**

To estimate the extent of problematic alcohol use in physicians and how it differs by physician sex, age, medical specialty, and career stage (eg, residency vs practicing physician).

**Evidence Review:**

Preferred Reporting Items for Systematic Reviews and Meta-analyses (PRISMA) 2020-compliant systematic review, searching Medline, Embase, and PsychInfo from January 2006 to March 2020. Search terms included Medical Subject Headings terms and keywords related to physicians as the population and problematic alcohol use as the primary outcome. The quality of studies was assessed using the Newcastle-Ottawa Scale. We included articles where problematic alcohol use was measured by a validated tool (ie, Alcohol Use Disorders Identification Test [AUDIT], AUDIT Version C [AUDIT-C], or CAGE [Cut down, Annoyed, Guilty, and Eye-opener] questionnaire) in practicing physicians (ie, residents, fellows, or staff physicians).

**Findings:**

Thirty-one studies involving 51 680 participants in 17 countries published between January 2006 and March 2020 were included. All study designs were cross-sectional, self-reported surveys. Problematic alcohol use varied widely regardless of measurement method (0 to 34% with AUDIT; 9% to 35% with AUDIT-C; 4% to 22% with CAGE). Reported problematic alcohol use increased over time from 16.3% in 2006 to 2010 to 26.8% in 2017 to 2020. The extent of problematic use by sex was examined in 19 studies, by age in 12 studies, by specialty in 7 studies, and by career stage in 5 studies. Seven of 19 studies (37%) identified that problematic alcohol use was more common in males than females. Based on the wide heterogeneity of methods for included studies, limited conclusions can be made on how problematic alcohol use varies based on physician age, sex, specialty, and career stage.

**Conclusions and Relevance:**

Studies about problematic alcohol use in physicians demonstrate a high degree of heterogeneity in terms of methods of measurement, definitions for problematic alcohol use, and cohorts assessed. Most studies are primarily self-reported, precluding the ability to determine the true prevalence among the profession. Few studies provide relevant comparisons to aid in identifying key risk groups for targeted interventions.

## Key Points

**Question:**

How common is problematic alcohol use among physicians, and what characteristics are associated with it in physicians?

**Findings:**

In this systematic review of 31 studies involving 51 680 participants in 17 countries, problematic alcohol use in physicians was identified by a self-reported survey, with reported use increasing over time. Methods of assessment and outcome definitions were highly variable, and limited information was identified on how problematic alcohol use varies among physicians based on age, sex, specialty, and training stage.

**Meaning:**

Key epidemiologic information of the prevalence of problematic alcohol use in physicians and associated risk factors are unknown, hampering the ability to identify high-risk individuals for targeted interventions.

## Teaser

This systematic review examines the extent of problematic alcohol use in physicians and how it differs by sex, age, medical specialty, and career stage in 31 studies from 17 countries.

## Introduction

Emerging evidence suggests physicians are at a higher risk of burnout and mental health conditions, including depression and anxiety, than the general population.^1,2,3,4,5,6,7,8^ Physicians are prone to occupational distress, which may facilitate problematic drinking habits, including drinking alcohol frequently, binge drinking, and alcohol use disorder.^9^ Although historical evidence suggests problematic alcohol use may be similar to those of the general population, this may be shifting over the last few decades with changes in the demographic composition of the physician workforce.^10,11,12,13,14,15^

Identifying problematic alcohol use in physicians is difficult. Behaviors that may indicate problematic alcohol use in a physician may include changes in behavior from baseline, loss of reliability, frequent medical complaints, mood changes, and legal problems due to impaired driving.^10,16^ Physicians with problematic alcohol use may be high functioning, making the identification of potential impairment challenging.^17^ Furthermore, societal stigma and fear of reprisal from professional colleges for reporting or seeking care for problematic alcohol use may encourage physicians with problematic alcohol use to keep their problems hidden.^18^

Given the long-term effects of alcohol on cognitive processes (including judgment, mood, impulse control, and learning), as well as health impacts (including cardiovascular disease, cancer, and liver cirrhosis), decreasing problematic alcohol use in physicians will improve physician health and well-being with the potential to improve patient care.^19,20,21,22^ Regarding patient care, problematic alcohol use has obvious and foreseeable clinical sequelae, such as an increase in physician error and absenteeism.^11,23^ As such, we conducted a systematic review of the literature to determine how common problematic alcohol use is reported by physicians and whether it differs by sex, age, specialty or career stage.

## Methods

### Protocol

This review followed an a priori protocol (PROSPERO CRD42022304799) developed and conducted according to the Preferred Reporting Items for Systematic Reviews and Meta-analyses (PRISMA) reporting guideline (Figure). We included peer-reviewed published studies or prepublication reporting on problematic alcohol use as measured with Alcohol Use Disorders Identification Test (AUDIT), AUDIT Version C (AUDIT-C), and the Cut down, Annoyed, Guilty, and Eye-opener (CAGE) questionnaire, among medical residents, fellows, and staff physicians that were published in English between January 2006 and March 2020. We excluded studies that (1) examined the prevalence of problematic alcohol use in medical students or nonphysician health care professionals (eg, nurses); (2) included both physicians and nonphysicians without reporting on both groups separately; (3) restricted data collection during major societal upheaval or crisis (eg, a war); or (4) were not original articles (eg, comments, letters, and reviews).

**Figure.**
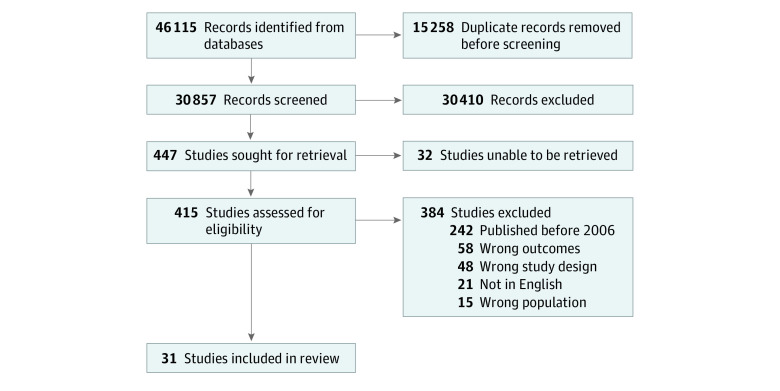
Flowchart of Study Selection

The primary outcome of interest in this study was the prevalence of alcohol use disorders or unhealthy alcohol use in this population, identified by standardized questionnaire, including the AUDIT, AUDIT-C, and the CAGE questionnaire. For this review, problematic alcohol use included hazardous, potentially hazardous, risky, at-risk, harmful, problematic, or heavy drinking or alcohol use, as well as alcohol misuse, alcohol dependence, and alcohol use more than low-risk guidelines and alcohol use disorder. Further detailed descriptions of the AUDIT, AUDIT-C, and CAGE questionnaires, including their structure, sensitivity, specificity, and what contexts they have been validated in, are included in eAppendix 1 in the Supplement 1. Details on the search strategy, data selection, and extraction and quality assessment are provided in eAppendix 2 in Supplement 1. Because there was a high degree of heterogeneity in the methods of measurement and definitions used, data synthesis (ie, meta-analysis and meta-regression) were not conducted.

## Results

### Study Characteristics

This review included 31 cross-sectional studies,^24-48^ involving a total of 51 680 medical residents and physicians across 17 countries. The characteristics of all studies can be found in Table 1.

**Table 1.** Design, Method of Measurement, and Outcomes for Included Studies Assessing Alcohol Use in Physicians

Source	Location	Study design	Sample, No.	Response rate (%)	Outcome assessment	Definition of outcome	Outcome, No. (%)
Sorensen et al,^24^ 2015	Denmark	CSS	1943	49	AUDIT	Hazardous use: 8-15	Hazardous: 300 (15.4)
						Harmful use: 16-19	Harmful: 46 (2.4)
						Alcohol dependence: ≥20	
Patel et al,^25^ 2017	Fiji	CSS	36	83.7	AUDIT	Zone II (8 [7 in F]-15): alcohol use > low-risk guidelines	Zone II: 10 (27.8)
						Zone III (16-19): harmful and hazardous drinking	Zone III: 2 (5.6)
						Zone IV (≥20): alcohol dependence	Zone IV: 0 (0.0)
Axisa et al,^26^ 2020	New South Wales, Australia	CSS	59	88	AUDIT	Risky drinking: M: 8-15, F: 7-15	Risky or high risk: 12 (20)
						High-risk drinking: ≥16	
Tobias et al,^27^ 2019	Maranhao, Northeastern Brazil	CSS	317	NR	AUDIT	Alcohol misuse: >8	39 (12.3)
Srensen et al,^28^ 2016	Denmark	CSS	1943	49	AUDIT	Hazardous alcohol use: ≥8	346 (18.3)
Obadeji et al,^29^ 2015	Ado-Ekiti, Nigeria	CSS	122	90.4	AUDIT	Hazardous use: ≥5	Hazardous: 8 (6.6)
						Harmful use: score not defined	Harmful: 1 (0.8)
Issa et al,^30^ 2012	Nigeria	CSS	241	68.9	AUDIT	Hazardous use: ≥5	10 (4.1)
Aalto et al,^31^ 2006	Finland	CSS	1909	59.8	AUDIT	Heavy drinking: ≥8	276 (14.5)
Talih et al,^32^ 2016	Lebanon	CSS	118	38	AUDIT	Harmful or hazardous use: ≥8	7 (6)
Bazargan et al,^33^ 2009	California, US	CSS	763	41	AUDIT	Hazardous drinking: >8	43 (5.7)
Pedersen et al,^34^ 2016	Denmark	CSS	1841	46	AUDIT	Risky or hazardous alcohol use: ≥8	346 (18.8)
Fond et al,^35^ 2018	Metropolitan France	CSS	2165	NR	AUDIT	Alcohol use disorder: M: ≥7, F: ≥6	736 (34.0)
Nash et al,^36^ 2010	Australia	CSS	2999	36	AUDIT	Potentially hazardous drinking: ≥8	438 (14.6)
Rosta and Aasland,^37^ 2010	Norway and Germany	CSS	2500	67.2	AUDIT in Norway; AUDIT-C in Germany	Hazardous drinking: ≥5	524 (21.0)
Wurst et al,^38^ 2013	Salzburg, Austria	CSS	456	18.6	AUDIT and AUDIT-C	At-risk drinking: AUDIT-C >5, AUDIT >8	AUDIT-C: 159 (34.9)
							AUDIT: 61 (13.4)
Sebo et al,^39^ 2007	Switzerland	CSS	1784	65	AUDIT-C	Hazardous drinking: M: >5, F: >4	533 (30)
Romero-Rodriguez et al,^40^ 2019	Spain	CSS	1331	6.4	AUDIT-C	Hazardous drinking: M: >5, F: >4	486 (27.8)
Oreskovich et al,^41^ 2015	US	CSS	7288	26.7	AUDIT-C	Alcohol abuse or dependence: M: ≥5, F: ≥4	1100 (15.1)
Oreskovich et al,^42^ 2012	US	CSS	7197	28.7	AUDIT-C	Alcohol abuse and possible dependence: M: ≥5, F: ≥4	1112 (15.4)
Lamberti et al,^43^ 2017	Naples, Italy	CSS	500	100	AUDIT-C	Hazardous alcohol consumption: M: ≥4, F: ≥3	43 (8.6)
Lebares et al,^44^ 2018	US	CSS	566	10	AUDIT-C	Hazardous drinking: M: ≥4, F: ≥3	194 (34.3)
						Alcohol abuse: M: ≥5, F: ≥4	131 (23.1)
Rosta,^45^ 2008	Germany	CSS	1917	58	AUDIT-C	Hazardous drinking: ≥5	380 (19.8)
Albano et al,^46^ 2020	Italy	CSS	639	NR	AUDIT-C	Hazardous drinking: M: ≥4, F: ≥3	58 (9.1)
Dyrbye et al,^47^ 2012	US	CSS	7197	28.7	AUDIT-C	At-risk drinking: M: >5, F: >4	984 (15.8)
Joos et al,^48^ 2013	Belgium	CSS	1501	6.1	AUDIT and CAGE	Hazardous drinking (AUDIT): M: >8, F: >6	Hazardous drinking: 270 (18.0)
						Screen positive for alcohol abuse (CAGE): ≥2	Problematic alcohol use: 275 (18.3)
McBeth et al,^49^ 2008	US	CSS	2397	56	CAGE	Screen positive for alcohol abuse: ≥2	133 (5.6)
Unrath et al,^50^ 2012	Germany	CSS	790	38.6	CAGE	Screen positive for alcohol abuse: ≥2	790 (18.9)
Rath et al,^51^ 2015	US	CSS	436	40.1	CAGE	Screen positive for alcohol abuse: ≥2	60 (15)
Mikalauskas et al,^52^ 2018	Lithuania	CSS	220	NR	CAGE	Screen positive for alcohol abuse: ≥2	48 (22)
Vetter et al,^53^ 2018	US	CSS	374	21.4	CAGE	Screen positive for alcohol abuse: ≥2	64 (17)
Pjrek et al,^54^ 2019	Austria	CSS	131	32.8	CAGE	Screen positive for alcohol abuse: ≥2	5 (3.8)

Abbreviations: AUDIT indicates Alcohol Use Disorders Identification Test; AUDIT-C, Alcohol Use Disorders Identification Test Version C; CAGE, Cut down, Annoyed, Guilty, and Eye-opener; CSS, cross-sectional survey; F, female; M, male; NR, not reported.

Our search of Medline, Embase, and PsycInfo yielded 30 857 records. After screening titles and abstracts, 447 were deemed eligible for full-text review. Of these, 32 studies were unable to be retrieved, 242 were published prior to January 2006, 58 lacked a clear outcome definition (eg, a validated questionnaire was not used), 48 were not original articles (eg, comments, letters, and reviews), 21 were not in English, and 15 included mixed populations (eg, health care workers without separate data reported for physicians). In total, 31 studies satisfied the inclusion and exclusion criteria of this study.

The number of participants in each study ranged from 36 to 7288 (median, 790; mean, 1667). Sixteen studies took place in Europe (3 in Denmark, 2 each in Austria, Germany, and Italy, 1 each in Belgium, Finland, France, Lithuania, Switzerland, and Spain, and 1 in Norway and Germany), 8 in North America (all in the US), 2 in Australia, 2 in Africa (both in Nigeria), and 1 each in South America (Brazil), Asia (Lebanon), and Oceania (Fiji). Eight studies^25,40,42,44,49,50,51,53^ included participants from a single specialty, 7 studies^27,33,34,37,38,43,46^ did not report the specialties of their participants, and 16 studies^24,26,28,29,30,31,32,35,36,39,41,45,47,48,52,54^ included participants from a variety of specialties. Twelve studies^25,27,28,29,31,33,38,42,47,50,51,53^ included fully trained physicians only (either reported this directly or this was assumed because the study reported time in practice), 7 studies^26,30,32,35,43,44,49^ included residents only, 7 studies^34,37,39,46,48,52,54^ did not report the career stage of included participants, and 5 studies^24,36,40,41,45^ included physicians in varying career stages.

The primary outcome was identified via self-report in all studies. No population-based studies using routinely collected health data were identified. The questionnaire used to identify problematic alcohol use was the CAGE in 7 of 31 studies,^48,49,50,51,52,53,54^ the AUDIT in 16 of 31 studies,^24,25,26,27,28,29,30,31,32,33,34,35,36,37,38,48^ and the AUDIT-C in 11 of 31 studies.^37,38,39,40,41,42,43,44,45,46,47^ Three studies used more than 1 questionnaire.^37,38,48^

The cut-off for what constituted problematic alcohol use varied between studies using the AUDIT and AUDIT-C. Additionally, 12 studies^25,26,35,39,40,41,42,43,44,46,47,48^ used different scoring cut-offs for problematic drinking based on sex, whereas 19 studies^24,27,28,29,30,31,32,33,34,36,37,38,45,49,50,51,52,53,54^ did not.

Studies using the AUDIT commonly used a cut-off of greater or equal to 8, but some used greater or equal to 7 or 6. The AUDIT questionnaire is well validated, with high sensitivity, and lower but still acceptable specificity for problematic alcohol use, although rates vary depending on the cut-off score used to identify a positive screen of a total of 40 possible points.^55,56^ A previous study^57^ demonstrated that among those diagnosed as having hazardous or harmful alcohol use, 92% had an AUDIT score of 8 or more, and 94% of those with nonhazardous consumption had a score of less than 8. Sensitivity varies between 97% for hazardous use, 95% for harmful drinking, and 51% to 59% for at-risk or heavy drinking. Specificity varies between 78% for hazardous use, 85% for harmful use, and 91% to 96% for at-risk heavy drinking.^57,58^

Most studies^37,38,39,40,41,42,44,45,47^ using the AUDIT-C used a cut-off of greater than 5 or 4. A cut-off of 4 or more has a sensitivity of 86% and specificity of 72% in identifying patients with heavy drinking and/or active problematic alcohol use or dependence.^59^

Studies^48,49,50,51,52,53,54^ using the CAGE questionnaire all were consistent, with a score of greater than or equal to 2 constituting a positive screen for alcohol abuse. The CAGE has demonstrated a mean (SD) sensitivity of 71% and specificity of 90% in varied samples of patients.^60^ The scoring criteria for each study can be found in Table 1.

### Extent of Problematic Alcohol Use Among Physicians

The reported extent of problematic alcohol use in physicians varied widely across all studies. The proportion of a positive screen varied between 0% to 34% for studies^24,25,26,27,28,29,30,31,32,33,34,35,36,37,38,48^ using the AUDIT, 8.6% to 34.9% for studies^37,38,39,40,41,42,43,44,45,46,47^ using the AUDIT-C, and 3.8% to 22.0% for studies^48,49,50,51,52,53,54^ using the CAGE questionnaire. The response rates varied between 6.1% to 100%, and 4 studies^27,35,46,52^ did not report a response rate. (Tables 1, 2, and 3).

**Table 2.** Studies Assessing Alcohol Use in Physicians by Age and Sex

Source	Location	Male, No. (%)	Female, No. (%)	Outcome assessment	Definition of outcome	Outcome by sex	Age distribution, No. (%), y	Outcome by age, y
Romero-Rodriguez et al,^40^ 2019	Spain	653 (37.1)	1107 (62.9)	AUDIT-C	Hazardous drinking: M, >5; F, >4	M, 222 (34.2); F, 264 (24.0)	NA	NA
Axisa et al,^26^ 2020	New South Wales, Australia	15 (25.0)	44 (75.0)	AUDIT	Risky drinking: M, 8-15; F, 7-15; high-risk drinking: ≥16	Risky or high risk: M, 0; F, 12 (27)	NA	NA
Srensen et al,^28^ 2016	Denmark	927 (47.7)	1016 (53.3)	AUDIT	Hazardous alcohol use: ≥8	M, 214 (23.1); F, 132 (13.0)	20-40, 557 (20.9); 41-50, 449 (16.8); 51-60, 564 (21.1); ≥61, 302 (11.3)	20-40, 103 (18.5); 41-50, 59 (13.1); 51-60, 113 (20.0); ≥61 y, 71 (23.5)
Obadeji et al,^29^ 2015	Nigeria	74 (61)	47 (0.39)	AUDIT	Hazardous use: ≥5; harmful use: score not defined	Hazardous use: M, 8 (10.8); F, 0; harmful use: M, 1 (1.4); F, 0	NA	NA
Lamberti et al,^43^ 2017	Naples, Italy	208 (41.6)	292 (58.4)	AUDIT-C	Hazardous alcohol consumption: M, ≥4; F, ≥3	M, 15 (7.2); F, 28 (9.6)	NA	NA
Sorensen et al,^24^ 2015	Denmark	927 (47.7)	1016 (53.3)	AUDIT	Hazardous use: 8-15; harmful use: 16-19; alcohol dependence: ≥20	AUDIT 8-15: M, 185 (21.7); F 115 (12.6); AUDIT ≥16: M, 29 (3.4); F, 17 (1.8)	20-40, 557 (28.7); 41-50, 453 (23.3); 51-60, 564 (29.0); ≥61, 373 (19.2)	AUDIT 8-15: mean (SD), 46.5 (12.5); 20-40, 91 (17.7); 41-50, 46 (10.7); AUDIT ≥16: mean (SD), 46.7 (12.6); 20-40, 12 (2.4); 41-50, 13 (3.1)
Oreskovich et al,^42^ 2012	US	6079 (84)	1041 (14.4)	AUDIT-C	Alcohol abuse and possible dependence: M, ≥5; F, ≥4	M, 846 (13.9); F, 266 (25.6)	<35, 194 (2.7); 35-44, 1596 (22.2); 45-54, 2238 (31.1); 55-64, 2077 (28.9); ≥65, 1015 (14.1)	<35, 33 (17.0); 35-44, 307 (19.2); 45-54, 379 (16.9); 55-64, 288 (13.9); ≥65, 105 (10.3)
Unrath et al,^50^ 2012	Germany	551 (69.7)	239 (30.2)	CAGE	Screen positive for alcohol abuse: ≥2	M, 551 (20.5); F, 239 (15.1)	31-45, 142 (18.0); 46-60, 502 (63.5); >60, 146 (18.5)	31-45, 142 (13.4); 46-60, 502 (20.7); >60, 146 (17.8)
Albano et al,^46^ 2020	Italy	279 (43.7)	360 (56.3)	AUDIT-C	Hazardous drinking: M, ≥4; F, ≥3	Low risk: M, 162 (58.1); F, 139 (38.6); high risk: M, 22 (7.9); F, 36 (10.0)	46 (22-69)^a^	At or high-risk: 54.5 (26-69)^a^
Rosta and Aasland,^37^ 2010	Norway and Germany	Norway: 398 (66.2); Germany: 1173 (61.8)	Norway: 203 (33.8); Germany: 725 (38.2)	AUDIT in Norway; AUDIT-C in Germany	Hazardous drinking: ≥5	Germany: M, 325 (27.7); F, 50 (6.9); Norway: M, 129 (32.4); F, 20 (9.9)	27-44, 1676 (67); 45-65, 823 (33)	27-44: 311 (18.5); 45-65: 213 (25.9)
Joos et al,^48^ 2013	Belgium	800 (53.3)	701 (46.7)	AUDIT and CAGE	Hazardous drinking (AUDIT): M, >8; F, >6; screen positive for alcohol abuse (CAGE): ≥2	Hazardous drinking: M, 166 (20.7); F, 105 (14.9); positive CAGE: M, 175 (21.9); F, 99 (14.1)	48.24 (12.7); <30, 87 (5.8); 30-44, 538 (35.2); 45-54, 356 (23.7); 55-64, 342 (22.8); >65, 164 (10.9)	Hazardous drinking: <30, 11 (12.6); 30-44, 77 (14.4); 45-54, 53 (14.8); 55-64, 70 (20.5); >65, 55 (33.7); positive CAGE: <30, 5 (5.7); 30-44, 82 (15.3); 45-54, 80 (22.5); 55-64, 71 (20.8); >65, 30 (18.3)
Nash et al,^36^ 2010	Australia	2098 (70)	868 (28.9)	AUDIT	Potentially hazardous drinking: ≥8	M, 366 (17); F, 72 (8)	<40, 485 (16.2); 40-49, 874 (29.1); 50-59, 924 (30.8); ≥60, 688 (22.9)	<40, 46 (9.5); 40-49, 145 (16.6); 50-59, 157 (17.0); ≥60, 90 (13.1)
Pjrek et al,^54^ 2019	Austria	73 (55.7)	58 (44.3)	CAGE	Screen positive for alcohol abuse: ≥2	M, 4 (5.5); F, 1 (1.7)	49.64 (6.74), 38-62; ≤49, 69 (52.7); ≥50, 62 (47.3)	≤49, 0; ≥50, 5 (8.1)
Issa et al,^30^ 2012	Nigeria	182 (75.5)	59 (24.5)	AUDIT	Hazardous use: ≥5	M, 10 (5.5); F, 0	NR	<34, 1 (10); 35-44, 7 (70); 45-54, 2 (20)
Aalto et al,^31^ 2006	Finland	712 (37.3)	1197 (62.7)	AUDIT	Heavy drinking: ≥8	M, 192 (27); F, 84 (7)	≤30, 214 (11.2); 31-40, 623 (32.6); 41-50, 708 (37.1); ≥51, 364 (19.1)	≤30, 32 (15.0); 31-40, 83 (13.3); 41-50, 88 (12.4); ≥51, 73 (20.1)
Wurst et al,^38^ 2013	Salzburg, Austria	244 (53.5)	204 (44.7)	18.6	AUDIT and AUDIT-C	≥8: M, 41 (16.8); F, 19 (9.3); ≥5: M, 91 (37.3); F, 32 (15.7)	NA	NA
Oreskovich et al,^41^ 2015	US	5191 (72.0)	2018 (28.0)	AUDIT-C	Alcohol abuse or dependence: M, ≥5; F, ≥4	M, 668 (12.9); F, 432 (21.4)	<35, 319 (4.4); 35-44, 1289 (17.7); 45-54, 1828 (25.1); 55-64, 2567 (35.2); ≥65, 1151 (15.8); missing, 55 (0.8)	<35, 68 (21.3); 35-44, 244 (18.9); 45-54, 297 (16.2); 55-64, 370 (14.4); ≥65, 117 (10.2)
Lebares et al,^44^ 2018	US	276 (49.1)	286 (50.9)	AUDIT-C	Hazardous drinking: M, ≥4; F, ≥3; alcohol abuse: M, ≥5; F, ≥4	Hazardous drinking: M, 110 (40); F, 168 (58); problematic alcohol use: M, 72 (26); F, 119 (41)	NA	NA
Rosta,^45^ 2008	Germany	1169 (61.0)	748 (39.0)	AUDIT-C	Hazardous drinking: ≥5	M, 327 (28.0); F, 53 (7.1)	NA	NA

Abbreviations: AUDIT indicates Alcohol Use Disorders Identification Test; AUDIT-C, Alcohol Use Disorders Identification Test Version C; CAGE, Cut down, Annoyed, Guilty, and Eye-opener; F, female; M, male; NA, not applicable; NR, not reported.

^a^ Median (range).

**Table 3.** Studies Assessing Alcohol Use in Physicians by Specialty Type and/or Career Stage

Source	Outcome assessment	Definition of outcome	Specialty distribution	Outcome by specialty	Stage distribution	Outcome by career stage
Rosta,^45^ 2008	AUDIT-C	Hazardous drinking: ≥5	Surgery, 492 (25.7); internal medicine, 561 (29.3); anesthesiology, 264 (13.8); obstetrics and gynecology, 136 (7.1); pediatrics, 100 (5.2); neurology, 65 (3.4); psychiatry and psychotherapy, 54 (2.8); radiology, 82 (4.3); urology, 56 (2.9); other, 105 (5.5)	Surgery, 113 (23.8); internal medicine, 97 (17.9); anesthesiology, 63 (24.8); obstetrics and gynecology, 20 (15.4); pediatrics, 8 (8.2); neurology, 9 (13.8); psychiatry and psychotherapy, 4 (7.7); radiology, 20 (24.7); urology, 18 (34.0); other, 11 (12.0)	NA	NA
Sorensen et al,^24^ 2015	AUDIT	Hazardous use: 8-15; harmful use: 16-19; alcohol dependence: ≥20	Emergency, 126 (6.9); general practice, 761 (41.7); occupational medicine, 31 (1.7); psychiatry, 92 (5.0); internal medicine, 337 (18.5); surgery, 172 (9.4); other, 305 (16.7); missing, 119	AUDIT 8-15: emergency: 23 (18.2); general practice, 110 (14.2); occupational medicine, 6 (18.6); psychiatry, 13 (14.3); internal medicine, 71 (21.4); surgery, 34 (20.0); other, 41 (12.7); AUDIT ≥16: emergency: 7 (5.6); general practice, 15 (1.5); occupational medicine, 1 (3.1); psychiatry, 2 (2.6); internal medicine, 8 (2.5); surgery, 3 (1.9); other, 9 (2.8)	Medical specialists and general practitioners: 1263 (68.9); junior doctors: 578 (31.3)	AUDIT 8-15, 16+; medical specialists and general practitioners: 204 (16.2), 31 (2.5); junior doctors: 96 (16.6), 15 (2.6)
Joos et al,^48^ 2013	AUDIT and CAGE	Hazardous drinking (AUDIT): M, >8, F, >6; screen positive for alcohol abuse (CAGE): ≥2	Surgery, 156 (10.4); anesthesia and reanimation, 116 (7.7); psychiatry and neurology, 208 (13.9); internal medicine, 267 (17.8); pediatrics, 141 (9.4); gynecology and obstetrics, 134 (8.9); others, 479 (31.9)	Hazardous drinking: surgery, 24 (15.4); anaesthesia and reanimation: 22 (19.0); psychiatry and neurology, 41 (19.7); internal medicine, 44 (16.5); pediatrics, 20 (14.2); gynecology and obstetrics, 31 (23.1); others, 88 (18.4); positive CAGE: surgery, 23 (14.7); anaesthesia and reanimation, 30 (25.9); psychiatry and neurology, 44 (22.1); internal medicine, 40 (15.7); pediatrics, 16 (11.3); gynecology and obstetrics, 29 (21.6); others, 88 (18.4)	NA	NA
Nash et al,^36^ 2010	AUDIT	Potentially hazardous drinking: ≥8	General practitioner, 590 (19.9); obstetrician and gynecologist, 179 (6.0); surgeon, 357 (12.0); anesthetist, 351 (11.8); psychiatrist, 231 (7.8); pathologist, 89 (3.0); radiologist, 107 (3.6); physician, 480 (16.1); accident or emergency specialist, 63 (2.1); pediatrician, 142 (4.8); in training, 254 (8.5); other, 128 (4.3); missing, 28	General practitioner, 73 (12); obstetrician and gynecologist, 27 (15); surgeon, 67 (19); anesthetist, 63 (18); psychiatrist, 35 (15); pathologist, 811 (12); radiologist, 16 (15); physician, 65 (14); accident or emergency specialist, 10 (16); pediatrician, 16 (11); in training, 33 (13); other, 22 (17)	NA	NA
Issa et al,^30^ 2012	AUDIT	Hazardous use: ≥5	Surgeons, 116 (48.1); general practitioner or medical officers, 10 (4.2); all other physicians, 115 (47.7)	Surgeons, 5 (4.3); all other physicians, 5 (4.3); general practitioner or medical officers, 0	NA	NA
Fond et al,^35^ 2018	AUDIT	Alcohol use disorder: M, ≥7; F, ≥6	Psychiatrists, 302 (13.9); other, 1863 (86.1)	Psychiatrists, 123 (40.7); other, 613 (32.9)	NA	NA
Romero-Rodriguez et al,^40^ 2019	AUDIT-C	Hazardous drinking: M, >5; F, >4	NA	NA	Physicians, 1330 (75.6); residents, 201 (11.4)	Physicians, 389 (29.4); residents, 50 (24)
Lebares et al,^44^ 2018	AUDIT-C	Alcohol misuse: M, ≥4, F, ≥3; alcohol abuse: M, ≥5, F, ≥4	NA	NA	Intern, 188 (33.3); PGY2, 104 (18.4); PGY3, 74 (13.1); PGY4, 62 (11.0); PGY5, 70 (12.4); lab, 66 (11.7)	Alcohol misuse; interns, 66 (41.25); PGY2, 45 (56.96); PGY3, 38 (61.29); PGY4, 21 (42.86); PGY5, 24 (42.11); lab, 33 (61.11); alcohol abuse; interns: 45 (28.13); PGY2, 28 (35.44); PGY3, 23 (37.10); PGY4, 16 (32.65); PGY5, 19 (33.33); lab, 23 (42.59)

Abbreviations: AUDIT indicates Alcohol Use Disorders Identification Test; AUDIT-C, Alcohol Use Disorders Identification Test Version C; CAGE, Cut down, Annoyed, Guilty, and Eye-opener; NA, not applicable.

### Differences in Problematic Alcohol Use in Physicians by Sex

Nineteen studies^24,26,28,29,30,31,36,37,38,40,41,42,43,44,45,46,48,50,54^ reported problematic alcohol use by sex (Table 2). Of these studies, the proportion of the male sample size varied between 25% and 75.5%. Problematic alcohol use was significantly higher in males than females in 7 studies and females than males in 4 studies. In general, recent studies (ie, published between 2015-2020) were more likely to report a female preponderance in problematic alcohol use. All (3 of 3) of the studies^41,42,44^ of physicians in the US reported higher rates of problematic alcohol use in females than males. One^43^ of 2 studies^43,46^ including Italian physicians showed that females were more likely to be at risk of high-risk drinking, while their male colleagues were more at risk of low-risk drinking, compared to no risk drinking. The other study^46^ in Italy showed that females were more likely to screen positive for hazardous alcohol consumption. Studies in the rest of Europe^24,28,31,37,40,45,48,50,54^ and Nigeria^29,30^ demonstrated that males were at a greater risk of screening positive for problematic alcohol use, while evidence in Australia was inconclusive.^26,36,38^

### Differences in Problematic Alcohol Use in Physicians by Age

Twelve studies^24,28,30,31,36,37,41,42,46,48,50,54^ reported problematic alcohol use by age (Table 2). All studies^24,28,30,31,36,37,41,42,48,50,54^ reported problematic alcohol use by age based on age groupings except for one study,^46^ which reported it as a median and range. Problematic alcohol use was higher in younger physicians in 2 studies and higher in older physicians in 2 studies. There were no significant differences in problematic alcohol use by age in 5 studies, and 3 studies did not report the statistical significance of their results.

### Differences in Problematic Alcohol Use in Physicians by Medical Specialty

Seven studies^24,30,35,36,41,45,48^ reported problematic alcohol use by medical specialty (Table 3). Five studies^24,36,41,45,48^ compared physicians across all specialties, while 2 studies^30,35^ compared problematic alcohol use in (1) surgeons vs general practitioners or medical officers vs all other physicians and (2) psychiatrists vs nonpsychiatrists. The extent of problematic alcohol use by medical specialty was similar in 5 studies. One study^45^ found that surgeons (including general surgery, obstetrics and gynecology, and surgical subspecialties) and anesthetists were significantly associated with hazardous drinking (OR, 1.4; 95% CI, 1.1-1.8; *P* < .001) compared with nonsurgical specialties (including internal medicine and subspecialties, pediatrics and psychiatry). Another study^41^ found that the prevalence of alcohol abuse or dependence was statistically significant (*P* = .001) between specialties, with the highest prevalence among dermatologists and orthopedic surgeons and the lowest prevalence among general pediatricians and neurologists.

### Differences in Problematic Alcohol Use in Physicians by Career Stage

Five studies^24,30,40,41,44^ reported problematic alcohol use by career stage (Table 3). One study^40^ found hazardous drinking was higher in practicing physicians vs residents (29.4% vs 24.0% based on an AUDIT-C score of >5 in males and >4 in females; *P* = .05). Another study^44^ used the AUDIT-C to assess alcohol misuse (≥4 in males and ≥3 in females) and alcohol abuse (≥5 in males and ≥4 in females) based on postgraduate year (PGY) of training. Rates of alcohol misuse increased significantly with the year of training (*P* = .011), while alcohol abuse varied.^44^ Another study^24^ compared the extent of hazardous (AUDIT 8-15) and harmful alcohol use (AUDIT 16-19) in medical specialists and general practitioners vs junior doctors with no significant difference detected (*P* = .754). One study^30^ reported that among hazardous users (AUDIT≥5), 30% were interns, 50% were residents, and 20% were consultants, but no indicators of statistical significance were reported. Although this study did not formally report outcomes based on the career stage, Oreskovich et al^41^ found that the extent of problematic alcohol use or dependence based on the AUDIT-C (≥5 in males and ≥4 in females) decreased significantly with years of practice (*P* < .001).

### Risk-of-Bias Assessment

Risk-of-bias assessment based on the Newcastle-Ottawa Risk-of-Bias Score found that 21 studies were graded as good quality, with 10 as poor (Table 4). All studies lost a point based on self-reported data. Most were penalized as not being representative of the target population (ie, a wide range of diverse physicians by specialty, sex, age, and career stage) or an unclear response rate (ie, less than 50%).

**Table 4.** Newcastle-Ottawa Scale Quality Assessment

Source	Selection (/5)	Comparability (/2)	Outcome (/3)	Overall study quality^a^
Sorensen et al,^24^ 2015	3		2	Poor
Patel et al,^25^ 2017	3	1	2	Good
Axisa et al,^26^ 2020	4	1	2	Good
Tobias et al,^27^ 2019	4	1	2	Good
Srensen et al,^28^ 2016	4	1	1	Poor
Obadeji et al,^29^ 2015	2	1	1	Poor
Issa et al,^30^ 2012	4		2	Poor
Aalto et al,^31^ 2006	4	2	2	Good
Talih, et al,^32^ 2016	3	2	2	Good
Bazargan et al,^33^ 2009	3	1	1	Poor
Pedersen et al,^34^ 2016	3	2	2	Good
Fond et al,^35^ 2018	3	2	2	Good
Nash et al,^36^ 2010	4	2	2	Good
Rosta and Aasland,^37^ 2010	3	2	1	Poor
Wurst et al,^38^ 2013	4		2	Poor
Sebo et al,^39^ 2007	4	1	2	Good
Romero-Rodriguez et al,^40^ 2019	4	1	2	Good
Oreskovich et al,^41^ 2015	4	2	1	Poor
Oreskovich et al,^42^ 2012	3	1	2	Good
Lamberti et al,^43^ 2017	5	2	2	Good
Lebares et al,^44^ 2018	4	2	2	Good
Rosta,^45^ 2008	4		1	Poor
Albano et al,^46^ 2020	4	2	2	Good
Dyrbye et al,^47^ 2012	3	2	2	Good
Joos et al,^48^ 2013	4	1	2	Good
McBeth et al,^49^ 2008	3	1	2	Good
Mikalauskas et al,^52^ 2018	4	2	2	Good
Vetter et al,^53^ 2018	3		1	Poor
Rath et al,^51^ 2015	3	1	2	Good
Unrath et al,^50^ 2012	4	2	2	Good
Pjrek et al,^54^ 2019	4	1	2	Good

^a^ Determined based on thresholds for converting the Newcastle-Ottawa scales to Agency for Healthcare Research and Quality standards. Good quality: 3 or 4 in selection domain and 1 or 2 in comparability domain and 2 or 3 in outcome or exposure domain. Fair quality: 2 in selection domain and 1 or 2 in comparability domain and 2 or 3 in outcome or exposure domain. Poor quality: 0 or in selection domain or 0 stars in comparability domain or 0 or 1 in outcome or exposure domain.

## Discussion

We conducted a systematic review to determine the extent of problematic alcohol use in physicians and identify high-risk groups or periods to inform screening and interventions. Overall, we identified 31 self-reported, cross-sectional, survey-based studies that reported the extent of problematic alcohol use in physicians. Most studies had low response rates, with only 4 of 31 studies exceeding 80%. Importantly, no population-based studies were identified, thereby limiting our understanding of the prevalence of problematic alcohol use in physicians.

AUDIT, AUDIT-C, and/or the CAGE questionnaire were most used to identify problematic alcohol; however, the definition of what constituted a positive screen for problematic alcohol use varied widely between studies (0%-34% using AUDIT, 8.6%-34.9% using AUDIT-C, and 3.8%-22.0% those using CAGE). In comparison, the prevalence of alcohol use disorder worldwide in 2019 was 1.45%, with prevalence rates highest in males aged 25 to 34 years.^61,62^ There is evidence to suggest that doctors are at an increased risk of anxiety and depression compared to the general population.^3,63,64^ Our results suggest that problematic alcohol use is also higher in physicians compared to the general population, although population-based studies with longitudinal designs or using health administrative data are needed to verify this trend.

We did observe an increase in the reported proportion of problematic alcohol use in physicians over the last 15 years from 16.3% to 26.8%. It remains unknown whether this increase is indeed accurate or whether it is due to increased transparency by physicians in self-reporting problematic alcohol use because of a changing culture of medicine.

The extent of problematic alcohol use by sex was examined in most (19 of 31) studies, and the largest proportion of these studies (7 of 19) reported a higher extent of problematic use in males than females. There were no clear differences in the extent of problematic alcohol use by age, physician specialty, and career stage. As such, key information on the extent of problematic alcohol use among physicians remains unknown.

Available data surrounding the extent of problematic alcohol use in physicians have historically come from license and disciplinary actions, known or registered problematic users, mortality rates, hospital admissions, and treatment populations, and surveys of selected groups of physicians.^12^ As these are highly select groups, the prevalence of problematic alcohol use in physicians remains unknown. Studies included in the current review are self-reported and are prone to biases limiting generalizability and accuracy. Self-reported alcohol consumption has been shown to amount to approximately 40% to 60% of total alcohol sales in the general population, which highlights the high likelihood that the extent on problematic alcohol use using self-reported data and is likely a vast underestimation of its true prevalence.^65,66^ Most studies reported low response rates suggesting physicians may be hesitant to participate in studies assessing problematic alcohol use. Physicians who use alcohol-related screening questionnaires as part of their practice may be familiar with the scoring systems and may answer in such a way as to screen negative for problematic alcohol use. Physicians may be likely to underreport use for fear of reprisal by colleagues and licensing boards. Therefore, the low levels of problematic alcohol use identified in this review likely underestimate the scale and consequent harms from alcohol use by physicians.

Periods of risk, specifically by age or career stage, that may increase one’s risk of problematic alcohol use were not identifiable. No differences in the extent of problematic alcohol use based on age was noted, suggesting that all age periods are equal risk or the heterogeneity and underreporting make identification of a true high risk age group difficult. Previous research suggests problematic alcohol use is higher in medical students than in practicing physicians, consistent with higher alcohol use in the general population.^67,68,69,70,71,72^ However, this may culturally based as Western countries are more likely to consume more alcohol in general.^73^ We were unable to identify differences in problematic alcohol use based on career stage, and it remains unclear whether career stage may influence a physician’s risk of problematic alcohol use.

In regard to sex-based differences, studies seem to report a male preponderance in problematic alcohol use, yet given the wide heterogeneity of the studies in terms of outcome reporting, quality of evidence, and geographical distribution, definitive conclusions are uncertain. Trends in drinking patterns in female physicians are likely driven by changing drinking patterns in women in general, suggesting that sex differences in drinking prevalence are converging.^74,75^ In the United States, the prevalence of high-risk drinking between 2001 and 2012 increased by 57.9% in women, relative to a 15.5% increase in men.^76^ We found geographic differences with female physicians in the US and Italy being more likely to screen positive for problematic alcohol use than men, whereas the converse was true in the rest of Europe and Nigeria. This observed geographic variability in sex differences appears consistent with the general population.^77^ Stress-related drinking has been noted to be a unique factor in alcohol use in women, and given the stressful nature of the profession of medicine, female physicians may be at an increased risk.^78^ Furthermore, the phenomenon of telescoping is more prevalent in female physicians than male physicians, as they are more likely to initiate alcohol use at a later age, but with shorter times from use to dependence and treatment.^79,80^ Female medical students may be more prone to developing problematic drinking habits throughout medical school, such that by the end of their training, rates of problematic alcohol use are similar between males and females.^67^

The identification of specialty-related differences in problematic alcohol use would also be very helpful to inform targeted screening for problematic alcohol use in physicians, workplace health promotion, and system-level change. Nonetheless, we found only 2 studies reported the extent of problematic alcohol use by specialty. These 2 studies reported surgical specialties are more likely to screen positive for problematic alcohol use relative to those in a nonsurgical specialty. Future research should aim to identify what specialties, including surgery, are associated with an increased risk of alcohol use and what environmental factors may be related.

Cultural changes minimizing stigma and reducing obstacles to seeking help may encourage physicians who suffer in silence to seek help. Future research could also aim to better understand factors that limit physician disclosure of problematic alcohol use and ultimately deconstruct these factors to promote care-seeking behavior in physicians. Furthermore, a clearer understanding of what sex, age, physician specialties, and career stages are most at risk for problematic alcohol use would help inform the development of physician health programs that identify problematic alcohol use and establish timely interventions for those in need.

### Limitations

This review has limitations. The primary outcomes of studies included in this review were very heterogeneous, which rendered comparison between studies quite challenging. We chose to include studies that reported on hazardous, potentially hazardous, risky, at-risk, harmful, problematic, or heavy drinking or alcohol use, as well as alcohol misuse, alcohol dependence, alcohol use more than low-risk guidelines, and alcohol use disorder. This was chosen to provide as comprehensive a picture as possible of the nature of problematic alcohol use in physicians. Nonetheless, some of these outcomes are discrete entities; for example, alcohol use more than low-risk guidelines is different from alcohol use disorder. We also did not select a specific cut-off for what constituted problematic alcohol use based on the AUDIT or AUDIT-C questionnaires and rather reported on the individual outcomes that were reported by each study however unstandardized they were. This made direct comparisons difficult and outlined the need for a large population-based study assessing the prevalence of problematic alcohol use based on an internationally accepted definition and standardized reporting. Furthermore, although this review included studies from across the globe, which increases the applicability and external validity of the review, cultural factors related to drinking patterns make it challenging to compare patterns of problematic alcohol use between countries. Lastly, given the number of articles retrieved in our initial literature search, we excluded articles that reported on binge drinking only. Nonetheless, binge drinking is not without its consequences and is generally considered to be a behavior indicative of problematic alcohol use and could contribute to physician impairment and poor patient outcomes.

## Conclusions

In this systematic review, we found that the prevalence of self-reported problematic alcohol use in physicians varied widely. All studies were survey-based and self-reported, with variable outcome definitions of problematic alcohol use and inconsistent reporting on differences across sex, age, physician specialty, and career stage. Future population-based studies with longitudinal designs or using health administrative data could help identify the prevalence of and salient risk factors for problematic alcohol use in physicians.

## Notes

Supplement. **eAppendix 1.** Additional Methodological Details **eAppendix 2.** Detailed Search Strategy

Supplement 2.
Data Sharing Statement
