# Supplementary material for: Characterization of Problematic Alcohol Use Among Physicians: A Systematic Review
Source: JAMA Netw Open. 2022 Dec 9;5(12):e2244679. doi: 10.1001/jamanetworkopen.2022.44679 (PMC9856419; doi:10.1001/jamanetworkopen.2022.44679)
Supplement: Supplement. — eAppendix 1. Additional Methodological Details eAppendix 2. Detailed Search Strategy [file jamanetwopen-e2244679-s001.pdf]

Supplemental Online Content

Wilson J, Tanuseputro P, Myran DT, et al. Characterization of problematic alcohol use among physicians: a systematic review. *JAMA Netw Open*. 2022;5(12):e2244679. doi:10.1001/jamanetworkopen.2022.44679

eAppendix 1. Additional Methodological Details

eAppendix 2. Detailed Search Strategy

This supplemental material has been provided by the authors to give readers additional information about their work.

© 2022 Wilson J et al. *JAMA Network Open.*

eAppendix 1. Additional Methodological Details

Detailed description of standardized questionnaires used to define problematic alcohol use

The AUDIT questionnaire was developed by the World Health Organization (WHO) in 1989 as a method to briefly assess and screen for hazardous and harmful patterns of alcohol use internationally in primary care.[81] The AUDIT has been used extensively in population health research, particularly in the primary care setting. It consists of ten questions that assess the domains of hazardous alcohol use (based on the frequency of drinking, typical quantity, and frequency of heavy drinking), symptoms of dependence (impaired control over drinking, impaired salience of drinking and morning drinking), and harmful alcohol use (guilt after drinking, blackouts, alcohol-related injuries, and concern from others about their drinking). The AUDIT is a valid tool to screen for problematic alcohol use in patients of diverse racial and ethnic backgrounds without bias.[82]

The AUDIT-C is a brief questionnaire that was developed in 1998 in the hopes of reducing the time burden of screening for heavy drinking, problematic alcohol use and dependence in the primary care setting.[28] It consists of three questions, all assessed on a 5-point Likert scale: Within the past year, (1) How often did you have a drink of alcohol? (2) How many standard drinks containing alcohol did you have on a typical day? (3) How often did you have six or more drinks on one occasion? The AUDIT-C has been shown to be effective in screening for alcohol misuse in multiple race/ethnic groups, demonstrating high Areas under the receiver operating curve (AUROCs) and similar specificities in White, African American and Hispanic subgroups. Differences sensitivities were not consistent in men and women, although the lowest sensitivity and highest specificity were found in the African American subgroup.[83]

© 2022 Wilson J et al. *JAMA Network Open.*

The CAGE questionnaire was developed by Dr. John Ewing and published in 1984.[84] It involves four questions that can easily be remembered by the acronym CAGE, consisting of: Have you ever (1) felt the need to cut down your drinking; (2) felt annoyed by criticism of your drinking; (3) had guilty feelings about drinking; and (4) taken a morning eye-opener? Each question is scored as 1 for 'yes' or 0 for 'no', and a score of 2 or greater is considered clinically significant. Limitations of the CAGE questionnaire include poor performance in white women, prenatal women and college students.[29,85,86]

The average response time for the AUDIT is ~ 78-131 seconds, and the CAGE questionnaire ~14-31 seconds.[87,88] The time to administer the AUDIT-C has not been assessed but would likely be slightly longer than the CAGE questionnaire, given the complexity of the questions. The CAGE and AUDIT questionnaires have been reported to have a correlation coefficient of 0.62 ($p < 0.01$), with a difference in internal consistency reliability of 0.14 (CAGE = 0.69, AUDIT = 0.83).[88] In comparison to the full AUDIT, the AUDIT-C has been shown to be superior for the detection of heavy drinking (AUROC = 0.891 vs. 0.881, $p = 0.03$), but inferior for the detection of active problematic alcohol use or dependence (AUROC = 0.811 vs. 0.786, $p < 0.001$).[28]

Search Strategy

We searched Medline (OVID interface), EMBASE (OVID interface) and PsychInfo (OVID interface) (see detailed Search strategy). The search initially included articles published between January 1990 and March 2020. Upon review of the number of articles retrieved by the search, articles before January 2006 were excluded to focus on more contemporary studies. Articles published after March 2020 were excluded to remove those that highlighted the burden of the COVID-19 pandemic on healthcare professionals. The search strategy was developed with the

© 2022 Wilson J et al. *JAMA Network Open.*

assistance of a health science librarian with expertise in systematic reviews and can be found in Supplementary Materials.

Data Selection and Extraction

Articles pulled from the databases were uploaded into the Covidence Systematic Review Management (Covidence, Melbourne, VIC, Australia) software, which removed duplicates. All titles and abstracts were screened by two independent screeners (two of JW, SN, SD, JH, PT) and categorized as eligible, ineligible, or possibly eligible. Any conflicts were resolved by a third reviewer. Full texts were retrieved and reviewed for articles marked as eligible or possibly eligible. Full inclusion and exclusion criteria can be found in the PRISMA flow diagram (Figure 1). Following full-text screening, two reviewers (two of JW, SN and JH) independently extracted data from eligible full-text articles, including publication details (author, title, year of publication, journal, etc.), details regarding the study population (sample size, country, specialty, etc.), outcome information, measures of association, covariates, and information related to missing data. Following data extraction, reviewers compared extracted data and consolidated the information into a final datasheet.

Quality Assessment

All included studies were compared to international reporting guidelines to ensure their validity. Risk of bias was evaluated using the Newcastle-Ottawa Scale (NOS), a quality assessment tool for non-randomized studies. This tool uses a 'star system' applied across three domains: study group selection, inter-group comparability, and exposure/outcome assessment. The study group selection section domain: (1) the representativeness of the sample in comparison to the target population, (2) whether the sample size was justified and satisfactory, (3) whether the response rate was satisfactory and comparability between respondents and non-respondent characteristics was

© 2022 Wilson J et al. *JAMA Network Open.*

established and (4) whether the exposure was ascertained using a validated vs. non-validated measurement tool. The inter-group comparability domain evaluates whether subjects in different groups were comparable and whether confounding factors were controlled. The exposure/outcome assessment domain evaluates: (1) how the outcome was assessed (e.g., independent blind assessment, self-report, etc.) and (2) whether the statistical test used to analyze the data was clearly described, appropriate and complete. Overall study quality was determined based on the thresholds for converting the Newcastle-Ottawa scales to Agency for Healthcare Research and Quality standards. Good quality is defined as 3 or 4 stars in the selection domain and 1 or 2 stars in the comparability domain and 2 or 3 stars in the outcome/exposure domain. Fair quality is defined as 2 stars in the selection domain and 1 or 2 stars in the comparability domain and 2 or 3 stars in the outcome/exposure domain. Poor quality is defined as 0 or 1 star in the selection domain or 0 stars in the comparability domain or 0 or 1 stars in the outcome/exposure domain. The assessment was completed by two independent reviewers (two of JW, PT and JH). Any conflicts were resolved by a third reviewer.

© 2022 Wilson J et al. *JAMA Network Open.*

eAppendix 2. Detailed Search Strategy

Completed July 20, 2021
Medline (OVID interface)
Date restriction: Limit ALL to dt=19900101-20200229

Physician Terms	Problematic Alcohol Use Terms
doctor*.ti,ab,kf. OR physician*.ti,ab,kf. OR resident*.ti,ab,kf. OR Physicians/ OR "Internship and Residency"/ OR Cardiologists/ OR Pulmonary Medicine/ OR Internal Medicine/ OR Pediatricians/ OR Gynecology/ or Obstetrics/ OR Orthopedic Surgeons/ or "Oral and Maxillofacial Surgeons"/ or Surgeons/ OR Psychiatry/ OR Dermatologists/ OR Endocrinologists/ OR Gastroenterologists/ OR Nephrologists/ OR Ophthalmology/ OR Pulmonologists/ OR Neurologists/ OR Radiologists/ OR Anesthesiologists/ OR Oncologists/ OR Neurosurgeons/ OR Allergists/ OR Physicians, Family/ OR Physicians, Emergency/ OR Pathologists/ OR Physiatrists/ OR Rheumatologists/ OR Urologists/ OR cardiologist*.ti,ab,kf. OR (Pulmonary adj Medicine).ti,ab,kf. OR (Internal adj Medicine).ti,ab,kf. OR Pediatrician*.ti,ab,kf. OR Gynecolog*.ti,ab,kf. OR Obstetrics*.ti,ab,kf. OR Surgeon*.ti,ab,kf. OR Psychiatr*.ti,ab,kf. OR Dermatologist*.ti,ab,kf. OR Endocrinologist*.ti,ab,kf. OR Gastroenterologist*.ti,ab,kf. OR Nephrologist*.ti,ab,kf. OR Ophthalmolog*.ti,ab,kf. OR Neurologist*.ti,ab,kf. OR Radiologist*.ti,ab,kf. OR Anesthesiologist*.ti,ab,kf. OR Oncologist*.ti,ab,kf. OR Neurosurgeon*.ti,ab,kf. OR Allergist*.ti,ab,kf. OR (Family adj Medicine).ti,ab,kf. OR Pathologist*.ti,ab,kf. OR Physiatrist*.ti,ab,kf. OR Rheumatologist*.ti,ab,kf. OR Urologist*.ti,ab,kf.	MESH Terms: alcohol-related disorders/ or alcoholism/ or binge drinking/<br><br>Keywords:<br>(alcohol adj1 abus*).ti,ab,kf. OR (alcohol adj1 addict*).ti,ab,kf. OR (alcohol adj1 dependen*).ti,ab,kf. OR (alcohol adj1 disorder*).ti,ab,kf. OR (alcohol adj1 use*).ti,ab,kf.

APA PsychInfo (OVID interface)
Date restriction: Limit ALL by YEAR – *end in 2020 and manually weed out Mar-Dec 2020*

Physician Terms	Problematic Alcohol Use Terms
doctor*.ti,ab. OR physician*.ti,ab. OR residen*.ti,ab. OR Physicians/<br><br>OR "Internship and Residency"/ OR Cardiologists/ OR Pulmonary Medicine/ OR Internal Medicine/ OR Pediatricians/ OR Gynecology/ or Obstetrics/ OR Orthopedic Surgeons/ or "Oral and Maxillofacial Surgeons"/ or Surgeons/ OR Psychiatry/ OR Dermatologists/ OR Endocrinologists/ OR Gastroenterologists/ OR Nephrologists/ OR Ophthalmology/ OR Pulmonologists/ OR Neurologists/ OR Radiologists/ OR Anesthesiologists/ OR Oncologists/ OR Neurosurgeons/ OR Allergists/ OR Physicians, Family/ OR Physicians, Emergency/ OR Pathologists/ OR Physiatrists/ OR Rheumatologists/ OR Urologists/ OR cardiologist*.ti,ab. OR (Pulmonary adj	MESH Terms: problematic alcohol use/ or "alcohol use disorder"/ or alcoholism/ or binge drinking/ or alcohol intoxication/<br><br>Keywords:<br>(alcohol adj1 abus*).ti,ab. OR (alcohol adj1 addict*).ti,ab. OR (alcohol adj1 dependen*).ti,ab. OR (alcohol adj1 disorder*).ti,ab. OR (alcohol adj1 use*).ti,ab.

© 2022 Wilson J et al. *JAMA Network Open.*

Medicine).ti,ab. OR (Internal adj Medicine).ti,ab. OR Pediatrician*.ti,ab. OR Gynecolog*.ti,ab. OR Obstetrics*.ti,ab. OR Surgeon*.ti,ab. OR Psychiatr*.ti,ab. OR Dermatologist*.ti,ab. OR Endocrinologist*.ti,ab. OR Gastroenterologist*.ti,ab. OR Nephrologist*.ti,ab. OR Ophthalmolog*.ti,ab. OR Neurologist*.ti,ab. OR Radiologist*.ti,ab. OR Anesthesiologist*.ti,ab. OR Oncologist*.ti,ab. OR Neurosurgeon*.ti,ab. OR Allergist*.ti,ab. OR (Family adj Medicine).ti,ab. OR Pathologist*.ti,ab. OR Physiatrist*.ti,ab. OR Rheumatologist*.ti,ab. OR Urologist*.ti,ab.	

EMBASE (OVID interface)
Date restriction: Limit ALL to dd=19900101-20200229

Physician Terms	Problematic Alcohol Use Terms
doctor*.ti,ab. OR physician*.ti,ab. OR residen*.ti,ab. OR Physicians/ OR "Internship and Residency"/ OR Cardiologists/ OR Pulmonary Medicine/ OR Internal Medicine/ OR Pediatricians/ OR Gynecology/ or Obstetrics/ OR Orthopedic Surgeons/ or "Oral and Maxillofacial Surgeons"/ or Surgeons/ OR Psychiatry/ OR Dermatologists/ OR Endocrinologists/ OR Gastroenterologists/ OR Nephrologists/ OR Ophthalmology/ OR Pulmonologists/ OR Neurologists/ OR Radiologists/ OR Anesthesiologists/ OR Oncologists/ OR Neurosurgeons/ OR Allergists/ OR Physicians, Family/ OR Physicians, Emergency/ OR Pathologists/ OR Physiatrists/ OR Rheumatologists/ OR Urologists/ OR cardiologist*.ti,ab. OR (Pulmonary adj Medicine).ti,ab. OR (Internal adj Medicine).ti,ab. OR Pediatrician*.ti,ab. OR Gynecolog*.ti,ab. OR Obstetrics*.ti,ab. OR Surgeon*.ti,ab. OR Psychiatr*.ti,ab. OR Dermatologist*.ti,ab. OR Endocrinologist*.ti,ab. OR Gastroenterologist*.ti,ab. OR Nephrologist*.ti,ab. OR Ophthalmolog*.ti,ab. OR Neurologist*.ti,ab. OR Radiologist*.ti,ab. OR Anesthesiologist*.ti,ab. OR Oncologist*.ti,ab. OR Neurosurgeon*.ti,ab. OR Allergist*.ti,ab. OR (Family adj Medicine).ti,ab. OR Pathologist*.ti,ab. OR Physiatrist*.ti,ab. OR Rheumatologist*.ti,ab. OR Urologist*.ti,ab.	MESH Terms: problematic alcohol use/ or "alcohol use disorder"/ or alcoholism/ or binge drinking/ or alcohol intoxication/<br><br>Keywords:<br>(alcohol adj1 abus*).ti,ab. OR (alcohol adj1 addict*).ti,ab. OR (alcohol adj1 dependen*).ti,ab. OR (alcohol adj1 disorder*).ti,ab. OR (alcohol adj1 use*).ti,ab.

© 2022 Wilson J et al. *JAMA Network Open.*
