# Supplementary material for: Characterization of Problematic Alcohol Use Among Physicians: A Systematic Review
Source: JAMA Netw Open. 2022 Dec 9;5(12):e2244679. doi: 10.1001/jamanetworkopen.2022.44679 (PMC9856419; doi:10.1001/jamanetworkopen.2022.44679)
Supplement: Supplement 2. — Data Sharing Statement [file jamanetwopen-e2244679-s002.pdf]

Data Sharing Statement

Wilson. Characterization of Problematic Alcohol Use Among Physicians: A Systematic Review. *JAMA Netw Open*. Published December 09, 2022. doi:10.1001/jamanetworkopen.2022.44679

Data
Data available: No

Additional Information
Explanation for why data not available: n/a
